# Emotional facial palsy: an unusual and rarely explored neurological sign

**DOI:** 10.1007/s10072-022-06256-9

**Published:** 2022-07-11

**Authors:** Giuseppina Miele, Luigi Lavorgna, Maria Maddalena Marrapodi, Gianmarco Abbadessa

**Affiliations:** 1 Department of Advanced Medical and Surgical Sciences, University of Campania “Luigi Vanvitelli”, Naples, Italy; 2 Department of Woman, Child and General and Specialist Surgery, University of Campania “Luigi Vanvitelli”, Naples, Italy

**Keywords:** Neuroanatomy, Cortico-facial tract, Emotional facial palsy, Clinical examination

## Abstract

The anatomy of the cortico-bulbar tract that drives voluntary movements of mimic muscles is well described. Some cases of facial palsy with inverse automatic-voluntary dissociation (emotional facial palsy; EFP) are reported in the literature. These cases suggested a completely independent path of the fibers whose lesion results in EFP. We aimed to review the clinical reports of EFP available in the literature to characterize the anatomical aspect of the fibers whose lesion results in the isolated impairment of spontaneous smiling. Cortico-pontine fibers that control spontaneous smiling arise from the medial surface of the prefrontal cortex and descend through the anterior limb of the internal capsule, thalamus, and brain steam, independently from those that control voluntary movement. The mesial temporal lobe, particularly the amygdala, plays a crucial role in the network driving emotionally evoked facial expressions. We would highlight the relevance of an unusual and rarely explored neurological sign that could be added to clinical examination in ruling out focal brain pathology, such as stroke, tumors, or multiple sclerosis.

## Introduction

Emotional facial paresis (EFP) is a rare condition characterized by the weakness of emotionally evoked facial movements. The clinical picture consists of a supranuclear hemifacial paresis exclusively in spontaneous smiling (inverse automatic-voluntary dissociation) [1].

Automatic-voluntary dissociation has been widely reported in stroke patients with damage to different motor areas [2]. Lesions in the territory of the middle cerebral artery (primary motor and pre-motor areas) compromise the ability to produce a symmetrical, voluntary smile, nevertheless without impairing the ability to smile in response to jokes [2].

These findings suggest an independent “limbic” pathway that mediates emotionally evoked facial movements.

Patients with ischemic damage involving the midcingulate cortex can make a voluntary smile, whereas they show the weakness of emotionally evoked facial movements. The emotional movements are controlled by the continuous inhibition of the voluntary motor centers, probably through the pyramidal fibers [3]. When a lesion occurs in these regions, voluntary movement is diminished, but emotional movements released from control become exaggerated on the “paralyzed” side [3]. A rapid evaluation can be performed in the outpatient setting to check the motor and emotional functions of the facial nerve. Any asymmetry in the resting position should first be noted by asking the patient to maintain an expressionless face. Next, he/she should be asked to exhibit various facial movements, such as raising the eyebrows and furrowing the forehead, forcefully closing the eyes, showing the teeth, protruding and clenching the lips as if to whistle and swelling the cheeks as if to inflate a balloon. The examiner should provoke a spontaneous smile by asking funny questions and telling hilarious stories during conversation to assess the emotional component of facial paresis.

Here, we aimed to review the clinical reports of EFP available in the literature to characterize the anatomical aspect of the fibers whose lesion results in the isolated impairment of spontaneous smiling.

## Supratentorial regions

EFP is often described as a sign of anterolateral thalamic infarction, secondarily to tubero-thalamic artery ischemia [3]. More rarely, EFP was observed due to lesions of the posterior thalamus [3]. The available literature also provides some reports of EFP as a result of ischemia in the contralateral midcingulate cortex and/or in the supplementary motor area (SMA), as well as a result of contralateral mesial temporal sclerosis (MTS) [4]. Patients with temporal lobe epilepsy and MTS frequently exhibited contralateral EFP, and some studies proposed this unusual neurological sign as a useful marker of lateralization of the damage [4]. Therefore, by virtue of its extensive connectivity to visual association areas in the temporal and frontal cortices, it was supposed that amygdala damage or disconnection could be crucial for emotionally evoked expression [5].

Moreover, lesions in deep frontal white matter, most tumors, sparing medial frontal cortex and thalamus result in EFP. The anterior and posterior parts of the thalamus are connected with the basal-temporal and frontal portion of the brain, and the presented clinical evidence suggests that these connections belong to the network that mediates emotionally evocated facial expression.

Trosch and colleagues [6] reported a patient with isolated EFP and striatum-capsular infarction without the involvement of the thalamus. The authors speculated that the sparing of the caudal quarter of the posterior limb of the internal capsule was responsible for the preservation of the volitional facial movements observed on this case [6]. This body of evidence supports the hypothesis that the EFP is realized due to the interruption of the connections between the thalamus and the medial-frontal and temporal lobes and that these connections lie in the anterior limb of the internal capsule.

## Upper brainstem

Furthermore, the EFP was found in the presence of brainstem lesions, even though more rarely [7–9]. Tumors in the subthalamic and dorsal mesencephalic region were described in patients presenting EFP, and it was attributed to the involvement of a small bundle of fibers within the dorsal midbrain segment near the midline [9]. The EFP due to pontine lesions has been related to the infarction of the dorsolateral pontine tegmentum, in line with the territory of the superior cerebellar artery [10]. The uniformity with which the EFP does not compare in the classic midbrain syndromes such as Benedikt’s or Weber’s syndrome and in the rostral pontine lesions is probably related to the ventral position of these lesions [3, 11]. Therefore, the cortico-pontine fibers responsible for the spontaneous smile would descend in the mediodorsal position in the midbrain and dorsal tegmentum in the pons separately by cortico-pontine fibers responsible for the volitional (or pyramidal) smile [8]. Further evidence supporting this hypothesis was the case of a multiple sclerosis patient [12] presenting a pattern of facial paresis consistent with a supranuclear lesion with concomitantly an unusual degree of preserved involuntary facial muscle innervation. The lesion in the ventral mesencephalon strengthens the hypothesis that, in the upper brain steam, fibers that control voluntary facial movements descend ventrally, whereas those that control emotionally evoked facial movements dorsally.

## Lower brainstem

Classically, a rostral lesion in the center of the upper pons causes a contralateral facial (volitional) supranuclear paresis, whereas the ipsilateral paresis of the infranuclear type derives from lesions of the inferolateral pons (VII nucleus).

Terao [2] and colleagues, examining the correlation of MRI brain stem lesions with neurological symptoms and signs, including central facial palsy, in 70 patients with localized infarction of the lower brainstem, hypothesized that the facial cortico-bulbar tract descends to the upper third of the medulla and, then, crosses and ascends towards the lower third of the pons reaching the nucleus of the VII cranial nerve.

Currier has suggested that the facial corticobulbar tract fibers leave the pyramidal tract at the pontomedullary junction and descend caudally, crossing to the opposite facial nucleus at the middle medullary level [13]. The interruption of these fibers above the decussation level has been suggested to result in a contralateral central facial palsy.

Therefore, the typical supranuclear facial paresis (contralateral to the side of the lesion) could be associated with the involvement of these descending pathways. Conversely, the involvement of the ascending fibers could be associated with central facial palsy homolateral to the side of the lesion.

In this regard, the only available report that described EFP due to lower brainstem lesion reported a case of EFP due to infarct in the territory of the anteroinferior cerebellar artery homolateral to the facial deficit [8]. The authors discussed that facial corticobulbar tract fibers descend caudally, independently from the pyramidal tract, up to the medulla’s upper third to reach the contralateral facial nucleus [2, 8].

Further, the results of Terao [2] and colleagues suggested that patients experienced central facial palsy more often when the lesion extends from the lower pons to the upper medulla than when it occurs in the middle to lower medulla. Inline Kim and colleagues [14] have also reported that, in patients with lateral medullary infarction, central facial palsy occurs more often with upper medulla than middle to lower medulla lesions. Overall, these findings suggest that the fibers descend at least to the level of the upper medulla, where they cross the midline.

The occurrence of an ipsilaterally EFP and an ischemic lesion in the territory of the lower anterior cerebellar artery, as in the case of Khurana and colleagues [8], may be representative of the involvement of the ascending part of the cortico-bulbar facial tract. It is believed that the corticobulbar facial fibers descend into the mediodorsal and basal portion of the pons, and the volitional and emotional fibers converge perhaps below the middle third of the pons. If the anatomical explanation of the ipsilateral EFP case is valid, this would imply that the fibers for emotions and volitional control follow a distinct path even after their decussation.

## Conclusions

In conclusion, cortico-pontine fibers that control spontaneous smiling arise from the medial surface of the prefrontal cortex and descend through the anterior limb of the internal capsule, thalamus, and brain steam, independently from those that control voluntary movement. The mesial temporal lobe, particularly the amygdala, plays a crucial role in the network driving emotionally evoked facial expressions. Therefore, EFP should be considered an unusual and rarely explored neurological sign that could be added to clinical examination in ruling out focal brain pathology, such as stroke, tumors, or multiple sclerosis.
